# Effects of orally administered hormonal contraceptives on the musculoskeletal system of healthy premenopausal women—A systematic review

**DOI:** 10.1002/hsr2.776

**Published:** 2022-08-10

**Authors:** Claudia Römer, Julia Czupajllo, Bernd Wolfarth, Markus H. Lerchbaumer, Kirsten Legerlotz

**Affiliations:** ^1^ Department of Sports Medicine, Charité—University Medicine Berlin Humboldt‐University of Berlin Berlin Germany; ^2^ Department of Radiology Charité—University Medicine Berlin, Humboldt‐University of Berlin Berlin Germany; ^3^ Movement Biomechanics, Institute of Sport Sciences Humboldt‐University of Berlin Berlin Germany

**Keywords:** adult, muscles, oral hormonal contraceptives, premenopausal women, tendons and ligaments

## Abstract

**Introduction:**

The musculoskeletal system (MSK) is one of the extragonadal target tissues of sex hormones: osteoblasts and osteocytes express estrogen receptors, while in fibroblasts of the anterior cruciate ligament (ACL) and myocytes of the vastus lateralis muscle (MVL), estrogen and progesterone receptors can be detected by immunoassay. Indeed, upon binding of sex hormones to the extragonadal receptors, the MSK seems to respond to varying levels of sex hormones with structural adaptation. Hormonal contraceptives can affect the musculoskeletal system; however, there is a lack of high‐quality studies, and no recommendation for female athletes exists.

**Material and Methods:**

This is a systematic review of publications on the effects of oral hormonal contraceptives on the biomechanical properties of tendons, muscles and ligaments, muscle strength, and soft tissue regeneration. A systematic database search was performed using MESH keywords and PRISMA (Preferred Reporting Items for Systematic Reviews and Meta‐Analyses) methodology in Pubmed and Cochrane to identify studies investigating the influence of oral hormonal contraceptives on muscles, tendons, and ligaments of healthy, adult, premenopausal women. The risk of bias in the studies included was assessed by two independent researchers using the ROBINS‐I Tool.

**Results:**

Nine comparative studies were identified that met the inclusion criteria. Endpoints were muscle strength and biomechanical tissue properties. No significant influence of oral hormonal contraceptives on muscle strength was found, although general muscle growth and Type I fiber growth were found to be significantly increased in a dose‐dependent manner. There was a negative effect on regeneration of muscle strength after exercise. The stiffness of tendons remained unchanged, while their size adaptation to load increased.

**Conclusion:**

The anabolic effect could be beneficial for specific sports, whereas reduced muscle regeneration could be disadvantageous for women exercising with high‐performance demands. The different effects on tendons and ligaments and the functional consequences of altered ligament and muscle stiffness, especially with regard to synthetic hormones, should be further investigated.

## 1 INTRODUCTION

The musculoskeletal system (MSK) is one of the extragonadal target tissues of sex hormones: osteoblasts and osteocytes express estrogen receptors, while in fibroblasts of the anterior cruciate ligament (ACL) and myocytes of the vastus lateralis muscle (MVL), estrogen and progesterone receptors can be detected by immunoassay.^1^, ^4^ Indeed, upon binding of sex hormones to the extragonadal receptors, the MSK seems to respond to varying levels of sex hormones with structural adaptation.^1^, ^4^ A higher estrogen concentration in the ACL was reported to be associated with a 40%–50% reduction in collagen synthesis and significantly reduced fibroblast proliferation^5^, ^6^ while exogenously administered estrogen after mechanical stress enhanced activation of satellite cells and proliferation of myoblasts in the MVL and in the soleus muscle in a mouse model.^7^ Furthermore, an inhibiting effect of oral contraceptives (OCs) on the synthesis of myofibrillary proteins in human muscles was detected.^8^ At once, hormonal contraceptives do not have an influence on the smooth muscle in arterial vessels and the menstrual cycle might influence endothelial function in major vessels.^9^, ^10^

Injury prevalence seems to vary through the menstrual cycle; however, results are inconclusive and the evidence is weak.^11^, ^14^ In this context, musculoskeletal and neurophysiological changes associated with varying hormone levels such as neuromuscular activation, joint laxity, postural control, or muscle strength are discussed as risk factors.^6^, ^11^, ^15^, ^16^ Anatomical differences between women and men also need to be considered, as a larger Q‐angle in women can also contribute to a higher ACL injury risk.^17^ Konopka et al.^18^ performed a systematic literature review for studies investigating the effect of OCs on the risk of soft tissue injuries and tissue laxity. While they identified 29 studies, only three were found to provide high‐level evidence. Overall, there are still conflicting findings in the current literature,^18^, ^21^ and well‐performed studies meeting the gynecological endocrinology criteria are lacking.^22^

Given that no evidence‐based recommendation exists regarding contraceptive methods for young female athletes, this systematic review was performed to address the research gap of OCs and their influence on the MSK with a focus on muscle strength and soft tissue changes and regeneration, which are important predictors for a higher risk of injury.^11^, ^12^

## 2 MATERIALS AND METHODS

### 2.1 Electronic database search

**Figure 1:**
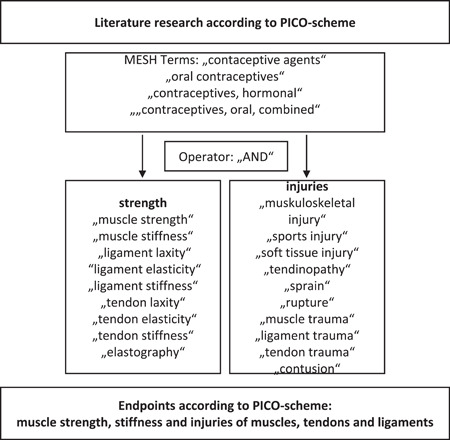
Flowchart of the research process in online database research. PICO, population, intervention, control, and outcomes.

In preparation of the literature search, we refined our research question in relation to population, intervention, control group, endpoints, and inclusion and exclusion criteria. The PICO (population, intervention, control, and outcomes) scheme was used for structuring the search process.^23^ Relevant studies were identified by an electronic literature search in PubMed and Cochrane online databases in June 2020. The search terms from the categories intervention and endpoints were combined as shown in Figure 1.

An exemplary search combination was accordingly “contraceptive agents” [MeSH Terms] AND “muscle strength” [MeSH Terms].” In the course of the search, each search term from the “Intervention” category was combined with each search term from the “endpoints” category. All search results in English and German language, regardless of the year, were considered and supplemented by manual searches in specialist journals. PRISMA (Preferred Reporting Items for Systematic Reviews and Meta‐Analyses) methodology was applied.

### 2.2 Inclusion and exclusion criteria

The inclusion and exclusion criteria for the selection of relevant studies were determined based on the PICO scheme as follows:

(1) In terms of the study population, we included studies that investigated healthy women from the age of 18 up to the onset of menopause. Exclusion criteria were studies of animal models, minors, postmenopausal women, or subjects not expressly defined as healthy.

(2) With regard to the intervention and control groups, studies were included in which a group of healthy women who took oral hormonal contraceptives in defined doses was compared with a female control group who did not use any hormonal contraceptives. Exclusion criteria were a lack of information on the dosage of the preparations taken, excessive doses, that is, an ethinyl estradiol (EE) content of more than 50 µg, hormonal contraceptives that were not administered orally, and a lack of a control group without taking hormonal contraceptives.

(3) With regard to the endpoints, studies were included that investigated the effects of oral hormonal contraceptives on the biomechanical properties of tendons, muscles and ligaments, muscle strength, and soft tissue regeneration within the groups compared. Studies that investigated other endpoints and, for example, dealt with the influence of oral hormonal contraceptives on bone density were excluded.

### 2.3 Selection process

**Figure 2:**
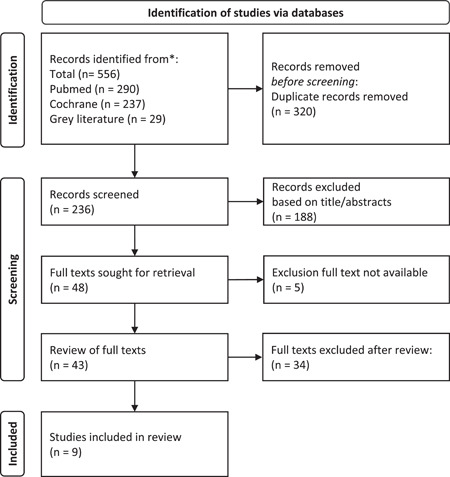
Selection process of the search results according to PRISMA (Preferred Reporting Items for Systematic Reviews and Meta‐Analyses) methodology

The systematic database search identified a total of 556 potentially relevant studies. The selection process is shown in Figure 2.

**Figure 3:**
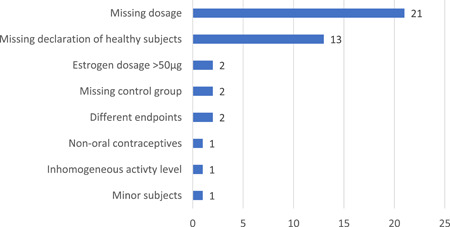
Reasons for exclusion after the full‐text screening

Reasons for study exclusion after full‐text screening are provided in Figure 3. Note that the high proportion of studies was excluded because no dose information on the contraceptives taken was provided.

Failure to explicitly state that the test subjects were healthy also led to numerous exclusions. The exclusion criterion of “inhomogeneous test group” (see Figure 2) relates to the diversity of physical activities in the study populations of Pokorny et al.,^21^ which could be a disruptive factor.

The risk of bias in the included studies was assessed by two independent researchers using the ROBINS‐I Tool^24^ and is shown in Table 4. For each study, the risk of bias was classified as low, moderate, serious, or critical. The risk of bias was assessed by taking into account confounders, participant selection, classifications of interventions, deviation from intended intervention, missing data, measurement, and result selection. Studies with >2 confounders were categorized as seriously biased. Measurement studies without any information on the investigators were classified as seriously biased.

## 3 RESULTS

### 3.1 Characteristics of the included studies

The publications included are comparative studies in which a group of test subjects taking OCs was compared with a group of test subjects who did not take hormonal contraceptives.^25^, ^33^ Ekenros et al.^3^ performed a cross‐over study, comparing phases with and without the intake of combined OCs (COCs). For this purpose, after the first measurement cycle, the subjects who were taking COCs at the start of the study stopped taking them and the subjects who were not using hormonal contraceptives at the start of the study started taking COCs at a dose comparable to that of the first group.^26^ Mackay et al.^31^ divided the control group according to the cycle phase into a subgroup in the follicular phase and a group in the ovulation phase. The endpoints of the included studies were muscle strength and/or properties of muscles, tendons, or ligaments; the level of significance was p < 0.05.

Table 1 lists important characteristics of the study populations. Table 2 summarizes the methodological characteristics and results of the studies identified to be relevant for the purpose of our literature review. In six of the studies, the subjects of the groups compared in the original studies had a further intervention; these are also shown in Tables 2 and 3.

**Table 1:** Characteristics of the study populations investigated in the studies included in our review

Study		Group	*N*	Age	BMI	Exercise activity	Cycle CG	EE dosage per Day in OC	PRG type and dosage per day in OC	OC intake
Dalgaard et al.^25^		OCG	14	24 ± 1	24 ± 1	<2 U/week	R	20–30 µg	75 µg (gestoden, *n* = 12);	Min. 1 year
									150 µg (desogestrel, *n* = 2)	
		CG	14	24 ± 1	23 ± 2					
Ekenros et al.^26^		OCG (bG)	8	Ø 26.4	Ø 23.7	2–3 SU/week	N/A	20–35 µg	150 µg (levonorgestrel, *n* = 10; desogestrel, *n* = 1);	N/A
									250 µg (norgestimat, *n* = 2);	
									500 µg (noretisterone, *n* = 1);	
									750 µg (lynestrenol, *n* = 1);	
									3 mg (drospirenone, *n* = 2);	
		CG (bG)	9	Ø 27	Ø 23.1					
Elliott et al.^27^		OCG	14	Ø 22	Ø 28.1	Not active	R	30–35 µg	150 µg (levonorgestrel, *n* = 8; desogestrel, *n* = 3);	Min. 6 months
									250 µg (norgestimat, *n* = 1);	
		CG	7	Ø 24	Ø 28.4				500 µg (noretisterone, *n* = 2);	
Hansen et al.^28^		OCG	15	22 ± 1	24 ± 1	High‐performance sports (handball)	R	20–35 µg	75 µg (gestoden, *n* = 5);	3–10 years
									150 µg (levonorgestrel, *n* = 1; desogestrel, *n* = 6);	
									3 mg (drospirenone, *n* = 3)	
		CG	15	23 ± 1	23 ± 1					
Lee et al.^29^		OCG	9	Ø 25.1	Ø 21.6	Not active	R	<50 µg	N/A	Min. 6 months
		CG	10	Ø 24.7	Ø 21					
Lee et al.^30^		OCG	15	Ø 25.1	Ø 22.3	≤2 SU/week	R	30–50 µg	N/A	Min. 1 year
		CG	25	Ø 25.2	Ø 21.9					
Mackay et al.^31^		OCG	10	26 ±3 .6	23.7 ± 4	Not active	R	20 µg	3 mg (drospirenone, *n* = 10)	N/A
		CG:	20:							
		FOL,	10,	29.4 ± 4.8	25.3 ± 2.9					
		OV	10	29.3 ± 6	23.5 ± 3					
Morse et al.^32^		OCG	12	20.3 ± 0.8	N/A	Not active	R	20–30 µg	3 mg (drospirenone, *n* = 12)	Min. 1 year
					Height: 165 ± 5 cm					
					Weight: 65.7 ± 1 kg					
		CG	12	19.8 ± 2.1	N/A					
					Height: 163 ± 5 cm					
					Weight: 64.7 ± 8.4 kg					
Romance et al.^33^		OCG	12	26.6 ± 3.7	22.3 ± 3	>2 years weight training, workload: N/A	R	30 µg	50 µg (gestoden, *n* = 8	Min. 6 months
		CG	11	28.3 ± 4.1	23.4 ± 2.2				150 µg (levonorgestrel, *n* = 4)	

Abbreviations: Ø, average; bG, before group change; BMI, body mass index; CG, control group; EE, ethinyl estradiol; FOL, subjects in follicular phase; FOV, subjects in ovulation phase; min., minimum; *N*, number of subjects; N/A, not specified; OC, oral contraceptives; OCG, group with OC intake; PRG, progesterone; R, regular; SE, sports units.

**Table 2:** Methodological characteristics of the included studies for muscle strength

Study	Study design	Results
Dalgaard et al.^25^	Intervention: 10‐week progressive resistance training program supervised by physical therapists	Overall: ‐ Significant strength gain (*p* < 0.001) with no significant group difference ‐ Anabolic effect at 30 µg EE (*p* = 0.01) ‐ Type I fiber growth increased in OCG (*p* < 0.05)
	Measurement methods: 1. Blood sample: ‐ Estradiol, progesterone 2. Biopsy: ‐ Vastus lateralis muscle (fiber analysis) 3. MRT: ‐ Cross‐sectional area of patellar tendon and vastus lateralis muscle in mm^2^ 4. RM: ‐ Knee extension in kg per kg bodyweight 5. Dynamometry: ‐ MVIC knee extension in Nm per kg bodyweight	Detailed: 1. Blood sample: ‐ Estradiol and progesterone in OCG < CG (*p* < 0.05) ‐ Estradiol is below the detection limit in 11 out of 14 OC users at baseline and 8 out of 14 OC users after the intervention 2. Biopsy: ‐ Type I fiber growth in OCG (*p* < 0.05), not in CG (*p* = 0.40) ‐ Type II fiber growth showed nonsignificant increase after the training period in both groups with no interaction between OC status and time (*p* = 0.11) 3. MRT: ‐ Anabolic effect on muscle growth with OC with 30 µg EE (*p* = 0.01) compared do CG, at 20 µg EE not significant compared to CG (*p* = 0.73) 4. RM and 5. Dynamometry: ‐ Strength gain in the overall group (*p* < 0.001) with larger absolute increases in the OCG but no significant group difference (RM: *p* = 0.46; MVIC: *p* = 0.36)
Ekenros et al.^26^	Intervention: Change of groups; measurement before and after the change of groups in different phases of the menstrual/OC cycle	Overall: no significant difference between groups
	Measurement methods: 1. Blood sample: ‐ Estradiol, progesterone 2. isokinetic measuring device (Biodex): ‐ MVIC knee extension; peak isokinetic muscle torque in Nm 3. Dynamometry: ‐ MVIC handgrip strength in kg 4. One‐legged jump: ‐ Distance of the hop from toe to heel in cm	Detailed: 1. Blood sample: ‐ Estradiol and progesterone in OCG < CG (*p* = not specified, data not shown) 2. Biodex ‐ No significant difference in knee extension MVIC after group change (*p* = 0.78) 3. Dynamometry: ‐ No significant difference in handgrip MVIC after group change (*p* = 0.76) 4. Jump: ‐ No significant difference in jump distance after group change (*p* = 0.78)
Elliott et al.^27^	Intervention: None; measurement in the luteal and follicular phase	Overall: No significant difference between groups
	Measurement methods: 1. Blood sample: ‐ Estradiol, progesterone 2. Dynamometry: ‐ MVIC of first dorsal interosseus muscle; force in N 3. isokinetic dynamometry: ‐ MVIC of the quadriceps and hamstring muscles in N	Detailed: 1. Blood sample: ‐ Estradiol and progesterone in OCG < CG (*p* < 0.05) ‐ No endogenous hormone fluctuation in OCG (*p* > 0.05) ‐ No significant correlation between estradiol or progesterone and any MVIC measure 2. Dynamometry: ‐ No significant group difference of first dorsal interosseus muscle MVIC in the luteal phase (*p* = 0.16) or follicular phase (*p* = 0.45) 3. Isokinetic dynamometry: ‐ Endpoint does not meet the inclusion criteria → not included (measurements only taken in OCG, not in the CG)
Mackay et al.^31^	Intervention: 30 min ergometer exercise at 90% of maximum concentric power output; Measurement before, immediately after and 48, 72, and 96 h after exercise	Overall: OC decrease muscle strength recovery (*p* = 0.01) and increase muscle pain (*p* < 0.01) after exercise, no differences in average power output between groups (*p* = 0.58)
	Measurement methods: 1. Saliva sample: ‐ Estradiol, progesterone 2. Blood sample (capillary): ‐ Creatine kinase 3. Leg press: ‐ MVIC knee extension in N 4. VAS: ‐ pain thigh muscles on a scale from 0 to 100 5. Algometer: ‐ pain threshold MVL in % of pre‐exercise value	Detailed: 1. Saliva sample: ‐ No significant group difference for estradiol (*p* > 0.05) at all measurements − 235% increase of progesterone at 96h in CG at OV, no increase in CG or OCG at the follicular phase ‐ No correlation between estradiol or progesterone with MVIC, creatine kinase, VAS, or pain threshold 2. Blood sample: ‐ Increase in creatine kinase activity in OCG > CG (*p* = 0.04). 3. Leg press: ‐ No significant group difference in MVIC strength (N) at baseline (*p* = 0.64). ‐ MVIC after 96 h in CG> OCG (*p* = 0.01) ‐ No recovery to baseline in OCG (*p* < 0.01). 4. VAS: ‐ No significant group difference at baseline (*p*> 0.05) ‐ After exercise OCG> CG (after 72 and 96 h: *p* < 0.01) ‐ in OCG no regeneration to baseline (*p* <0.01) 5. Algometry: ‐ In OCG no regeneration to baseline (*p* < 0.01)
Morse et al.^32^	Intervention: None	Overall: No significant group difference
	Measurement methods: 1. K100 electronic goniometer: ‐ Angle measurement of the ankle joint during passive foot dorsiflexion 2. Dynamometer: ‐ MVIC at plantar flexion in Nm	Detailed: 1. Electrogoniometry: ‐ Group difference (*p* > 0.05) 2. Dynamometry: ‐ Group difference (*p* > 0.05)
Romance et al.^33^	Intervention: 8‐week training program under defined nutrition	Overall: OC increase gain of fat‐free mass after training (*p* < 0.05), effect on strength gain (*p* > 0.05)
	Measurement methods: 1. DXA: ‐ Body mass in kg ‐ Fat mass in kg ‐ Fat‐free mass in kg 2. RM: ‐ Squats in kg ‐ Bench press in kg 3. Countermovement jump: ‐ Jumping power measured by jump height in cm	Detailed: 1. DXA: ‐ Group difference *p* > 0.05 for body mass, fat mass and fat‐free mass at baseline ‐ Significant increase in body mass and fat‐free mass in OCG (*p* < 0.05) but not in CG 2. RM: ‐ Significant increases in squat and bench‐press RM for both groups (*p* < 0.05) ‐ Group difference at baseline and after training program for squat and bench press RM (*p* > 0.05) 3. Countermovement jump: ‐ Effect of training program on countermovement jump (*p* > 0.05) ‐ Group difference in countermovement jump at baseline and after training program (*p* > 0.05)

Abbreviations: ATT, anterior tibial translation; CG, control group; DXA, dual‐energy X‐ray absorptiometry, cm, centimeters; EE, ethinyl estradiol; IGF‐1, insulin‐like growth factor; kg, kilograms; mm, millimeters; MRT, magnetic resonance tomography; MVIC, maximum voluntary isometric contraction (force); N, Newton; Nm, Newtonmeters; OC, oral contraceptives, OCG, group with OC intake; RM, repetition maximum (moving weight); US, ultrasound; VAS, visual analog scale.

**Table 3:** Methodological characteristics of the included studies for biomechanical properties

Study	Study design	Results
Dalgaard et al.^25^	Intervention: 10‐week progressive resistance training program supervised by physical therapists	Overall: ‐ No significant group differences in tendon quality
	Measurement methods: 1. Blood sample: Estradiol, progesterone 2. Biopsy: Patellar tendon (crosslinks) 3. MRT: Cross‐sectional area of patellar tendon and vastus lateralis muscle in mm^2^	Detailed: 1. Blood sample: ‐ see Table 2 2. Biopsy: ‐ Trend: Crosslinks in patellar tendon in OCG > CG (*p* = 0.07) 3. MRI: ‐ Cross‐sectional area of the patellar tendon was significantly increased compared to baseline (*p* < 0.05) with no difference between groups in the response to training
Hansen et al.^28^	Intervention: none; measurement in the menstrual phase and luteal phase	Overall: Greater cross‐sectional area in the patellar tendon of the jumping leg in the OCG (*p* = 0.05) → higher adaptation to load; inverse correlation of serum estradiol and patellar tendon stiffness in CG (*p* = 0.04)
	Measurement methods: 1. Blood sample: Estradiol, progesterone, IGF‐1 2. MRT: Length in cm and cross‐sectional area of patellar tendon in mm^2^ 3. US and dynamometry: Change in length/dislocation of the patellar tendon while increasing to MVIC → calculation of Patellar tendon stiffness from knee extension force/dislocation 4. Biopsy: Patellar tendon (collagen content in mg per mg dry weight, quantified by measuring hydroxyproline and crosslink parameters, quantified by measuring hydroxylysyl pyridinoline, lysyl pyridinoline, and pentosidine hydroxyproline)	Detailed: 1. Blood sample: ‐ Estradiol, progesterone, and IGF‐1 in OCG < KG (*p* < 0.05) 2. MRT: ‐ Larger patellar tendon cross‐sectional area in the jumping leg compared to contralateral patellar tendon (*p* = 0.09), effect correlates significantly with OC‐intake (*p* = 0.05) 3. Mechanical characteristics: ‐ No significant group difference in patellar tendon stiffness (*p* = 0.57) ‐ Inverse correlation between estradiol level and patellar tendon stiffness tendency in CG (*p* = 0.04) ‐ Moderate positive correlation in CG between estradiol level and dislocation (*p* = 0.09) as well as length variation (*p* = 0.06) of the patellar tendon 4. Biopsy: ‐ No significant group difference in patellar tendon collagen content (hydroxyproline, *p* = 0.25) and crosslinks (hydroxylysyl pyridinoline, *p* = 0.48; lysyl pyridinoline, *p* = 0.69; pentosidine hydroxyproline *p* = 0.94)
Lee et al.^29^	Intervention: Heat application of 38°C at the knee joint and quadriceps femoris muscle; measurement in the menstrual, luteal, follicular, and ovulatory phase	Overall: OC increases the ligament stiffness (*p* < 0.05) and the force required to move the knee (*p* < 0.05), heat application reduces fluctuations in the stiffness of the anterior crucial ligament in CG and reduces the force required to move the knee (*p* < 0.05)
	Measurement methods: 1. Blood sample: Estradiol 2. KT‐2000 arthrometer: ATT in mm 3. Electronic goniometer and motorized movement splint: force used to flex and extend the knee in N	Detailed: 1. Blood sample: ‐ Estradiol fluctuation during the menstrual cycle in CG (*p* < 0.001) no significant fluctuation in the OC cycle (*p* = 0.42) 2. Arthrometry: ‐ ATT OCG < CG, regardless of temperature (*p* < 0.05) ‐ Significant variation of ATT during the menstrual cycle in CG at room temperature (*p* < 0.01), after heat application (*p* = 0.44) ‐ In OCG no variation of ATT during the OC cycle (room temperature: *p* = 0.89, heat application: *p* = 0.97) 3. Measurement of strength: ‐ Expended strength at room temperature and heat application OCG > CG (*p* < 0.05) ‐ Expended strength decreases after heat application: in CG in menstrual phase (*p* = 0.04) und follicular phase (*p* = 0,01); in OCG significant in all phases (*p* < 0.05)
Lee et al.^30^	Intervention: 15 min of squats; measurement at 24, 48, and 72 h postexercise	Overall: OC increase ligament stiffness (*p* = 0.01) before exercise, no significant effect of OC on the extent of the ligament stiffness increase after exercise, pain in OCG > CG
	Measurement methods: 1. VAS: pain in the thigh muscles on a scale from 0 to 10 2. KT‐2000 arthrometer: ATT in mm	Detailed: 1. VAS: ‐ Pain after exercise OCG > CG (*p* = 0.008) with a pain peak 24 h postexercise in CG and 48 h postexercise in OCG ‐ No significant difference in VAS between 48 and 72 h postexercise in both groups (*p* > 0.05) 2. Arthrometry ‐ ATT in baseline OCG < CG (*p* = 0.01) ‐ Lower ATT in all participants postexercise with lowest values in OCG ‐ Significant decrease in ATT in the overall group at 48 h postexercise (*p* = 0.02) ‐ No significant group variation in ATT decrease (*p* > 0.05)
Morse et al.^32^	Intervention: none	Overall: OC decrease passive muscle stiffness (*p* < 0.01)
	Measurement methods: 1. K100 electronic goniometer: angle measurement of the ankle joint during passive foot dorsiflexion 2. US: Length and dislocation of the gastrocnemius medialis muscle–tendon unit under passive stretching in cm	Detailed: 1. Electrogoniometry: ‐ Group difference (*p* > 0.05) 2. US: ‐ Passive muscle stiffness OCG < CG (*p* < 0.05); greater displacement of the muscle–tendon unit at all torque angles during passive dorsiflexion

Abbreviations: ATT, anterior tibial translation; CG, control group; DXA, dual‐energy X‐ray absorptiometry, cm, centimeters; EE, ethinyl estradiol; IGF‐1, insulin‐like growth factor; kg, kilograms; mm, millimeters; MRT, magnetic resonance tomography; MVIC, maximum voluntary isometric contraction (force); N, Newton; Nm, Newtonmeters; OC, oral contraceptives; OCG, group with OC intake; RM, repetition maximum (moving weight); US, ultrasound; VAS, visual analog scale.

Table 4 summarizes the risk of bias classifications for all studies included. The risk was assessed using the ROBINS‐I Tool.^24^ More detailed information on the risk assessment procedure can be found in the Supporting Information: Material.

**Table 4:** Risk of bias classification of the nine studies included in the systematic review using the ROBINS‐I Tool according to Sterne et al.^24^

Study		Bias domain							Comments
		I	II	III	IV	V	VI	VII	
Dalgaard et al.^25^		L/M	L	L	L	M	L/M	L	I: No control for cycle phase; large range of OC‐intake duration; lower protein intake/kg body weight in CG compared to OCG V: Muscle fiber CSA was evaluated on 9/14 in OCG and 7/14 in CG VI: Specific information on examiners (number, qualification, knowledge of intervention status) is only given for 3/5 endpoints
Ekenros et al.^26^		M	L/M	L	L	M	S	L	I: No information for OC‐intake duration; no information on diet and activity during the study II: Inclusion/exclusion criteria are not clearly listed V: It is not clearly written/shown in the result text or graphs if data were successfully obtained for all subjects VI: No information on examiners (number, qualification, knowledge of intervention status)
Elliott et al.^27^		L	L/M	L	L	L	S	L	II: Inclusion/exclusion criteria are not clearly listed VI: No information on examiners (number, qualification, knowledge of intervention status)
Elliott et al.^28^		L	L	L	L	L	L/M	L	I: Thorough evaluation of potential confounders VI: Information on examiners considering blinding only given for 2/3 of endpoints
Lee et al.^29^		S	M	L	L	L	M	L	I: vague information on activity level; no information on diet and smoking status; no information on how long no OC was taken in CG; no specification of OC type and progesterone dosage; large BMI range (15–30) including clinically over‐ and underweight subjects II: No information about the recruitment process VI: No information if the examiners were blinded
Lee et al.^30^		S	L	L	L	M	M	L	I: No information on diet and smoking status; no consideration of cycle phases; no presentation of OC type and progesterone dosage; large BMI range (15–30) II: No information about the recruitment process V: It is not clearly written/shown in the result text/graphs if data were successfully obtained for all subjects VI: No information if the examiners were blinded
Mackay et al.^31^		M	M	L	L	L	S	L	I: No information on diet, smoking status, comedication and duration of OC intake II: No information about the recruitment process, limited inclusion/exclusion criteria VI: No information on examiners (number, qualification, knowledge of intervention status)
Morse et al.^32^		S	M	L	L	M	S	L	I: No information on health status except for lower extremity injury as exclusion criterium; no information on comedication, smoking status or diet; no consideration of menstrual/OC cycle phases II: No information about the recruitment process, inclusion/exclusion criteria not listed V: It is not clearly written/shown in the result text/graphs if data were successfully obtained for all subjects VI: No information on examiners (number, qualification, knowledge of intervention status)
Romance et al.^33^		L	L	L	L	L	M	L	VI: No information on blinding of examiners but supervision of all testing sessions by the research team

Abbreviations: I, confounding; II, participant selection; III, classification of interventions; IV, deviation from intended intervention; V, missing data; VI, measurement; VII, result selection; BMI, body mass index; C, critical risk of bias; CG, control group; CSA, cross‐sectional area; L, low risk of bias; M, moderate risk of bias; OC, oral contraceptives; OCG, group with OC intake; S, serious risk of bias.

### 3.2 Effect on hormonal fluctuation

Several studies consistently showed that COC intake significantly reduced estrogen and progesterone levels and their fluctuations.^25^, ^29^, ^31^

### 3.3 Effects on muscle strength

Six studies investigated the influence of COCs on muscle strength.^25^, ^27^, ^31^, ^33^ Overall, they neither showed a significant effect on muscle strength nor on its increase through a training program.^25^, ^27^, ^30^, ^31^, ^33^ However, Dalgaard et al.^25^ and Romance et al.^33^ showed an anabolic effect on general muscle growth as well as on Type I fibers, while Mackay et al.^31^ detected a negative effect of COCs on regeneration of muscle strength after exercise and muscle soreness. Creatine kinase increased with COC intake, which correlated with greater pain.^31^, ^34^ However, a severe risk of bias for measurement was found in four studies.^26^, ^27^, ^31^, ^32^ Overall, female athletes may use COCs with an estrogen dose of <50 µg without suffering from any disadvantages in terms of strength performance.

### 3.4 Effects on the texture and elasticity of muscles, tendons, and ligaments

Five studies investigated the influence of COCs on morphology and mechanical properties of muscles, ligaments, and tendons.^25^, ^28^, ^30^, ^32^ The stiffness of the patellar tendon was not significantly affected by COCs.^28^ Structural differences were only detected by Dalgaard et al.,^25^ with a higher content of crosslinks in tendons in COC‐taking women. There were no significant group differences in collagen content.^28^ Hansen et al.^5^, ^8^ showed reduced insulin‐like growth factor‐1 messenger RNA or protein expression during COC intake. The tendon cross‐sectional area correlated significantly with COC intake.^28^ The ACL was generally more rigid during COC intake; however, less markedly so after warm‐up.^29^ Lee et al.^29^, ^30^ did not observe a significant influence of COC on the increase in ACL stiffness after strong mechanical loading. During passive stretching, stiffness of the gastrocnemius medialis muscle was significantly reduced in all angles in women taking COCs.^32^

## 4 DISCUSSION

The aim of this literature search was to present the current state of scientific evidence on the influence of COCs on muscles, tendons, and ligaments of young healthy women. In nine selected comparative studies, a population of a total of 232 young, healthy women with different levels of physical activity was investigated.^25^, ^31^ Overall, COCs did not significantly affect muscle strength regardless of the test subjects' exercise load, despite a dose‐dependent anabolic effect on general muscle growth and especially Type I fiber growth^25^, ^27^, ^31^, ^33^ and a potentially reduced muscle stiffness.^28^, ^30^, ^32^

### 4.1 Musculoskeletal injury risk and OCs

ACL stiffness was increased when COCs were taken, as was the cross‐sectional area increase of the patellar tendon in response to loading.^28^, ^30^ However, no significant differences in patellar tendon stiffness were reported.^28^, ^30^

Whether the musculoskeletal adaptations observed in women taking COCs affect the risk of injury remains to be established and should be investigated in future studies including a larger number of athletes.^18^

A previous systematic review investigating the effect of OCs on injury risk found that the results of the studies included were inconsistent.^18^ For the endpoint of injury risk, two of their included studies rated with a high level of evidence suggested a protective effect of OCs on ACL injuries.^18^ The protective effect may be related to changes in collagen turnover, as Hansen et al.^28^ detected a reduced collagen turnover in women taking COCs and reduced collagen synthesis in the patellar tendon in women taking OCs.^35^ The constellation of increased muscle growth without a measurable increase in strength found in the present work is consistent with Thomas et al.,^7^ who reported induction of satellite cell activation and myoblast proliferation by estrogen administration in a mouse model. Likewise, an inhibitory effect of OCs on the synthesis of myofibrillar proteins was described by Hansen et al.^8^

### 4.2 Influence of OCs on muscle and tendon stiffness

Although none of the studies included in our review had the occurrence or risk of MSK injuries as endpoint, the publications discuss possible consequences of the effects of COCs on tissue stiffness.^29^, ^30^, ^32^ In the study of Hansen et al.,^28^ the estrogen level in the control group correlated inversely with patellar tendon stiffness. Across both groups, irrespective of OC intake, estrogen levels tended to correlate with deformation and strain of the patellar tendon, suggesting a higher risk of injury.^28^ Lee et al.^29^ also reported the fluctuation in ACL stiffness during the menstrual cycle to show an inverse correlation to the estrogen level. They assume the higher ACL stiffness observed in women taking COCs to potentially reduce the risk for ACL tears.^29^ Overall stiffness of the knee joint, measured as the force exerted to flex and extend the knee joint, was higher when COCs were taken.^29^ Ligament stiffness was lower when heat was applied, especially in the group taking COCs.^29^ Heat eliminated the inversely correlating fluctuation of ACL stiffness with the estrogen level, suggesting that the higher body temperature around ovulation may contribute to differences between OC and non‐OC users.^29^ Morse et al.^32^ point out that any change in muscle stiffness can have an impact on the muscle response to eccentric contractions, which in turn affects the risk of injury, although a possible role of COCs is not further discussed.

Lee et al.^29^, ^30^ described an increased ACL stiffness in women taking COCs. These results agree with the results of a systematic literature review conducted by Leblanc et al.^36^ ACL stiffness, measured as anterior tibial translation, was reduced in the analyzed studies under the influence of high estrogen levels.^36^ The discrepant effects of COCs on collagen synthesis and stiffness of the ACL and the patellar tendon should be investigated further in comparative studies of the hormonal effects on tendons and ligaments.^5^, ^6^ Furthermore, new MSK measurement tools, such as shear wave elastography and MyotonPro, should be considered for measuring MSK stiffness in future studies.^37^, ^38^

### 4.3 Soft tissue regeneration

The anabolic effect and the induction of Type I fiber growth could be a positive side effect of taking COCs.^25^, ^27^ Possible disadvantages of COC intake include poorer regeneration of muscle strength and longer‐lasting muscle soreness after exercise,^30^, ^31^ which is useful information physicians taking the history of female athletes should be aware of. In women with a high training volume, for example, in ambitious leisure or competitive sports, this aspect could be a limiting factor for training motivation and performance. Gynecologists and sports medicine physicians need to take this into account.^22^

### 4.4 Measurement methods

As exact reporting of the measurement procedure is essential for studies of the MSK, authors should both describe the measurement method and the examiners' experience. In terms of the measurement method, four of the studies included in our systematic review were found to be severely biased.^26^, ^27^, ^31^, ^32^ Romance et al.^33^ assessed muscle growth by determining the lean mass, a method that is prone to error due to changes in water content. Specifically, the determination of tissue stiffness and practicability of the measurement method turned out to be difficult.^33^ Lee et al.^29^, ^30^ investigated ACL stiffness by determining ATT using the validated KT‐2000 arthrometer, an examination that is carried out manually in everyday clinical practice using the so‐called Lachmann test, which does not allow precise quantification.^39^, ^40^ In contrast, Hansen et al.^8^ calculated patellar tendon stiffness from dynamometric and sonographic measurements, which is a far more direct but also far more time‐consuming method to measure ligamentous stiffness and thus challenging to integrate into clinical practice. Ultrasound elastography could offer a noninvasive and easy‐to‐use alternative measurement method here.^41^ While it is currently used in various specialist areas such as quantification of liver fibrosis or breast cancer detection, ultrasound elastography can also be applied to quantify the elasticity of muscles, tendons, and ligaments.^42^, ^44^ Another alternative is the MyotonPro, a portable device for the noninvasive measurement of mechanical tissue properties.^37^ It detects changes in tissue stiffness by emitting a mechanical impulse and recording the resulting tissue oscillation.^37^, ^45^

### 4.5 Differences in endogenous and synthetic hormones

It is well known that OCs affect the natural hormonal fluctuation.^46^ In addition, synthetic hormones, such as EE, do differ in receptor affinity compared to endogenous hormones,^47^ which needs to be considered in the research of athletes using COCs in comparison with naturally menstruating women.^22^ Differences in hormone profiles between COC users and non‐COC users in the examined studies were to be expected, as the cycle phases are suppressed in COC users, which results in a negative feedback reaction and a lower endogenous estradiol concentration.^22^ Furthermore, four studies included COCs with different progestins^25^, ^28^, ^33^ and two studies did not report the type of progestin,^29^, ^30^ limiting the interpretation of the results. Future studies need to consider a correct determination of the cycle phases and should examine COC users with the same preparation to avoid inconclusive results.^22^

### 4.6 Limitations

Consistent use of narrow inclusion and exclusion criteria with regard to population and intervention resulted in an overall homogeneous study population under precisely defined estrogen influence, which is quite representative in relation to the age group of users of OCs.^48^ In future studies, a restriction of progesterone content might be defined in the inclusion criteria, since progesterone effects on the MSK are less well understood compared with the estrogen and gestagen content of COCs.^25^, ^27^, ^49^ Overall, a small population size of the studies is a weakness. Another limitation was found for Lee et al.^30^ and Morse et al.,^32^ whose measurements were performed without considering the phase of the menstrual cycle and without measuring sex hormone levels. This is a disruptive factor given the strong fluctuations during the menstrual cycle.^10^, ^12^, ^22^, ^30^, ^32^ For future studies, it is fundamental to adhere to specific criteria, such as an exact definition of cycle phases, and a specialist in gynecological endocrinology should be involved in the study design.^22^ Numerous relevant publications had to be excluded because they provided no dosing information. In future work, the preparations taken as well as the duration of intake and the cycle history should be precisely surveyed and documented.^22^ These facts were missing in Ekenros et al.^26^ and Mackay et al.^31^

## 5 CONCLUSION

The studies included in this literature review reported no effect of COCs on muscle strength. However, it is well established that estrogen and progesterone levels and their fluctuations are significantly reduced in women taking COCs. Overall, the results of this systematic review suggest that possible disadvantages of COC intake include poorer regeneration of muscle strength and longer‐lasting muscle soreness after exercise. This aspect could be a limiting factor for training motivation and performance. Gynecologists and sports medicine physicians need to take this into account. For future work, there is a need for high‐quality studies with stricter application of existing, well‐defined gynecological criteria.

## AUTHOR CONTRIBUTIONS

Claudia Römer and Kirsten Legerlotz conceived the presented idea. Claudia Römer and Julia Czupajllo performed the literature review, final inclusion, and evaluation of the studies. Claudia Römer and Kirsten Legerlotz drafted the manuscript. All authors have read and approved the final version of the manuscript. Claudia Römer had full access to all of the data in this study and takes complete responsibility for the integrity of the data and the accuracy of the data analysis.

## CONFLICT OF INTEREST

Markus H. Lerchbaumer reports having received consultancy honoraria from Canon Medical Imaging and Siemens Healthineers. The remaining authors declare no conflict of interest.

## ETHICS STATEMENT

The systematic literature search was carried out in compliance with Charité's statutes aimed at ensuring good scientific practice.

## TRANSPARENCY STATEMENT

Claudia Römer affirms that this manuscript is an honest, accurate, and transparent account of the study being reported; that no important aspects of the study have been omitted; and that any discrepancies from the study as planned have been explained.

## Data Availability

The authors confirm that the data supporting the findings of this study are available within the article and its supplementary materials.
